# A Survey of Fatty Acid Content of the Male Reproductive System in Mice Supplemented With Arachidonic Acid

**DOI:** 10.1155/jl/3351340

**Published:** 2024-12-19

**Authors:** Viridiana Abigail Correa-Navarro, Gloria del Carmen Romo-Morales, Jaime Eduardo Sánchez-Palafox, Dalia Rodríguez-Ríos, Jorge Molina-Torres, Enrique Ramírez-Chávez, Silvio Zaina, Gertrud Lund

**Affiliations:** ^1^Department of Medical Sciences, Division of Health Sciences, Leon Campus, University of Guanajuato, 20 de Enero 929, Leon, Guanajuato, Mexico; ^2^Department of Genetic Engineering, CINVESTAV Irapuato Unit, Km 9.6 Libramiento Norte, Carretera Irapuato-León, Irapuato, Guanajuato 36824, Mexico; ^3^Department of Biotechnology and Biochemistry, CINVESTAV Irapuato Unit, Km 9.6 Libramiento Norte, Carretera Irapuato-León, Irapuato, Guanajuato 36824, Mexico

**Keywords:** arachidonic acid, fatty acid, male reproductive system, paternal transmission

## Abstract

Paternal exposure to high-fat diets or individual fatty acids (FAs) including arachidonic acid (AA) modifies progeny traits by poorly understood mechanisms. Specific male reproductive system FAs may be involved in paternal inheritance, as they can modify a range of cellular components, including the epigenome. Our objective was to determine FAs in compartments of the male reproductive system that potentially affect ejaculate composition—right and left testicular interstitial fluid (TIF), vesicular gland fluid (VGF), and epididymal adipose tissue (EAT)—in mice exposed to AA or vehicle daily for 10 days (*n* = 9–10/group). Whole blood (WB) and interscapular brown adipose tissue (IBAT) FA profiles were used as reference. AA significantly affected only VGF FAs relative to vehicle, that is, increased and decreased levels of arachidic and docosahexaenoic acid, respectively, versus vehicle (0.28% ± 0.01% and 0.23% ± 0.03%, respectively, *p* = 0.049, and 2.42% ± 0.47% and 3.00% ± 0.58%, respectively, *p* = 0.041). AA affected distinct FAs in WB. Additionally, we uncovered AA-dependent and AA-independent FA laterality. Myristic acid was higher in AA-exposed left versus right TIF (0.68% ± 0.35% and 0.60% ± 0.11%, respectively, *p* = 0.004). Right TIF contained higher oleic and linoleic acid and lower stearic acid than left TIF (29.01% ± 3.07% and 24.00% ± 2.18%, respectively, *p* = 0.005; 9.14% ± 1.88% and 7.05% ± 1.36%, respectively, *p* = 0.005; and 21.90% ± 2.92% and 26.01% ± 2.46%, respectively, *p* = 0.036), irrespective of exposure to AA. The TIF oleic/stearic acid ratio suggested higher Stearoyl-CoA Desaturase 1 activity in the right versus the left testis (1.35 ± 0.32 and 1.00 ± 0.17, respectively, *p* = 1.0 × 10^−4^). Multitissue comparisons revealed that TIF and VGF FA profiles were distinct from WB, EAT, or IBAT counterparts, suggesting tissue-specific FA fingerprints. In conclusion, AA modulated selected VGF long-chain FAs that may impact on uterine inflammation and subsequent embryonic development. AA altered local FA synthesis or selective uptake, rather than eliciting passive uptake from WB. Additionally, we uncover a significant laterality of testis FAs that may result in asymmetric sperm cell phenotypes.

## 1. Introduction

The 20-carbon omega-6 polyunsaturated fatty acid (PUFA) arachidonic acid (AA) is highly relevant from a nutritional and biological viewpoint in humans. AA can be synthesized from the essential fatty acid (FA) linoleic acid or can be obtained from the diet, mainly from eggs, chicken, and fish [1]. Additionally, breastmilk is an important source of dietary AA [2]. AA is the precursor of eicosanoids that both mediate and resolve inflammation: prostaglandins, leukotrienes, and thromboxane [3, 4]. The dysregulated synthesis of those factors is associated with impaired immune response and increased risk for a plethora of diseases. The latter includes cardiovascular disease, obesity, and cancer [5–8]. On the other hand, AA is crucial for infant development (reviewed in [9]).

We have shown that paternally or maternally supplemented AA exerts intergenerational effects in mice. Paternal AA supplementation for three generations induces cumulative metabolic effects. Along the paternal line, that is, founder and three successive paternally exposed generations, AA induced an increase in body weight in the last generation. Furthermore, the cumulative supplemented paternal AA dose correlated with several organ weights including liver. In the liver, milligrams of maternal and paternal AA supplement correlated positively with global DNA methylation and negatively with Stearoyl-CoA Desaturase 1 (*Scd1*) promoter methylation. Consistent with the involvement of SCD1, paternal and maternal AA dose was directly associated with liver *cis*-hexadecenoic acid, an anti-inflammatory omega-9 isomer of palmitoleic acid that is synthesized by SCD1 [10]. In a separate study, the effect of paternally supplemented AA on progeny's behavioural phenotype was addressed following exposure to lipopolysaccharide (LPS). Paternal AA primed progeny for behaviour consistent with increased anxiety in a sex-specific manner. In particular, high AA doses mimicked LPS exposure in males. Again underlining the relevance of SCD1, high AA doses interacted with LPS by modulating the expression of that enzyme in the hypothalamus [11]. Consistent with the above data, paternally supplemented fish oil, a surrogate of specific FA, ameliorated the metabolism of the progeny in a mouse model [12, 13]. The data imply that molecular information transmitted via the sperm induces a memory of an individual's paternal exposure to AA. This is in essence the concept of paternal intergenerational inheritance, which has been shown in a variety of metabolic and behavioural models [14, 15]. The nature of the sperm-borne molecular information that determines paternal inheritance is poorly understood. That information can be carried by the sperm cell or by other components or the ejaculate. For example, a high-fat diet alters specific sperm cell noncoding RNAs that impact transcription in the zygote and potentially in the foetus and postnatally [16]. Additionally, nongenetic inheritance mechanisms based on transmission of FA-rich cellular structures have been proposed [17]. Additionally, the vesicular gland plays a role in inter- and transgenerational metabolic effects following paternal exposure to a low-protein or high-fat diet in rodents [18–21]. Likewise, a study in invertebrates has highlighted a role of seminal fluid in “transgenerational immune priming”—that is, the transfer of a cellular memory of immune exposure from fathers to offspring [22]. A further layer of complexity is that paternal exposure can generate changes in other components of the male reproductive system that, in turn, signal the sperm. Those include the germline-hosting testicular environment and the epididymal adipose tissue (EAT). EAT may produce signals that affect the surrounding male reproductive system akin to the regulation of vascular tissue inflammation by the adjacent pericardial or perivascular fat [23, 24]. In particular, the tight proximity of EAT to the testis suggests the possibility that proinflammatory factors are transferred from EAT to the testis either by diffusion or through local vasculature as proposed for perivascular fat [25].

Relatively few studies have addressed the role of sperm FA in intergenerational effects. High-fat diet exposure in male mice imposes a proinflammatory omega-3/omega-6 FA ratio in the sperm of grandsons [26]. The short FA valproic acid affects the sperm DNA methylation [27]. Broadly, the case for lipids in intergenerational epigenetic inheritance has been presented [28]. FA may be both the signal and the mark of AA-induced intergenerational effects. As signals, AA or its metabolite(s) may alter the transcriptional program of the sperm cell, given that FA and circulating lipids can modify DNA methylation and the chromatin in several eukaryotic models and in humans [29–34]. Additionally, AA may induce a mark consisting of an ejaculate FA pool that affects the uterine wall or the zygote. In accordance with those hypotheses, peroxisome proliferator–activated receptors (PPARs) mediate the intergenerational effects of paternal traumatic stress or cadmium exposure in mouse models, and selected FAs are well-characterized PPAR ligands [35, 36].

In the light of the above considerations, we determined total FA in both the right and left testicular interstitial fluids (TIFs) separately, in the vesicular gland fluid (VGF) and the EAT—in mice exposed to AA or vehicle daily for 10 days. The rationale for surveying the TIF was that it reflects the FA composition of the testicular environment, thus representing a readout of FA profile that the germline is exposed to [37]. We analyzed left and right TIF separately, as gonadal asymmetry has been appreciated in biology, human medicine, and art since Classical Greece [38–41]. In particular, the right testis has been reported to be heavier than the left counterpart in mice, pointing to nonrandom but strain-specific asymmetric determinants of growth and development [42]. To our knowledge, the mechanism and functional consequence of testicular asymmetry have not been addressed. As FAs are associated with organ weight, we speculate that testis FA, whether modified by AA supplementation, may show laterality and provide preliminary hints on mechanisms. Whole blood (WB) and interscapular brown adipose tissue (IBAT) FA profiles were used as reference, to assess interorgan similarities of FA pools and to what extent passive intake from WB determines organ FA. We discuss the data in the light of the current knowledge on the bioactivity of FA and paternal intergenerational effects.

## 2. Materials and Methods

### 2.1. Animal Supplementation

The protocol was approved by the Institutional Committee for Ethics in Research of the University of Guanajuato (CEPIUG) with Approval No. P44-2022. Mice were fed chow (LabDiet No. 5001) and had free access to water. Twelve-week-old C57BL/6 male mice were orally supplemented with a daily dose of 1.2 *μ*g AA (98.5% pure, Sigma–Aldrich Cat. No. A3611) mixed with soybean oil (vehicle; Nutrioli) in a total of 5 *μ*L or vehicle alone for 10 days (*n* = 10/group). Two to six mice for each of five litters were randomly assigned to either experimental group, and each litter was equally represented in the two groups. The vehicle has no detectable AA and contains 0.01% tert-butylhydroquinone, an antioxidant that should prevent AA chemical modification (the FA composition of vehicle previously obtained by us is shown in Table S1 [10]). Additionally, vehicle contains the physiological AA precursor linoleic acid at a concentration of 7.5 g per serving (15.4 mL) (http://www.nutrioli.com) or a maximum of 2.4 mg per 5-*μ*L supplement. Vehicle linoleic acid is expected to modestly impact the final endogenous concentration of AA, as supplemented linoleic acid contributes to tissue AA one order of magnitude less than supplemented AA (reviewed in [9].) Furthermore, dietary linoleic acid levels are not associated with tissue AA [43]. The AA dose was chosen based on our previous study that demonstrated intergenerational effects of AA [10, 11]. That dose represents 0.45% of total FA (or 0.05% of total daily energy) and is within values recommended for infant nutrition and reflects actual dietary patterns [1, 44]. AA or vehicle was supplemented by gently depositing the 5-*μ*L solution into the side of the mouth with a blunted pipette tip as previously reported [10].

### 2.2. Tissue Obtention and Processing

Mice were sacrificed by decapitation under isoflurane anaesthesia. The WB was obtained during decapitation. The testis, vesicular gland, IBAT, and EAT were dissected, and subsequent tissue manipulation was performed in ice. The TIF was obtained from each testis separately (i.e., left and right TIF) as previously reported [37]. Briefly, each testis was decapsulated by careful removal of the tunica albuginea, weighed, and immersed in the same volume of cold phosphate-buffered saline (PBS) (1:1 ratio between milligrams of decapsulated testis and milliliters of PBS) for 45 min to allow diffusion of the TIF. The mix of testis and PBS was centrifuged at 1000 × *g* for 5 min; the supernatant was recovered and cleared by centrifugation at 1660 × *g* for 15 min. The supernatant was carefully separated from the pellet to obtain the TIF. To isolate the VGF, each horn of the vesicular gland was cut into three segments and the VGF was carefully squeezed out with a spatula. The IBAT was identified and dissected as reported [45]. All samples were stored at −80°C until used.

### 2.3. Total FA Determination

Total FAs were determined from lyophilized 25–50-mg tissue or total VGF obtained from an individual mouse (variable volume), or 200 *μ*L WB or TIF, by gas chromatography–mass spectrometry as previously described [10].

### 2.4. Statistics and Data Visualization

To compare individual FA of the same tissue between AA-supplemented and control groups, percentage values were compared by the Mann–Whitney *U* test. To assess laterality, within-individual-mouse paired comparisons of right and left TIF were conducted for each individual FA using the Wilcoxon paired test. For all other multitissue comparisons of individual FA, overall significance was tested with the Kruskal–Wallis ANOVA followed by the Wilcoxon paired test. FA profiles (i.e., average percent values for each of the determined FAs) between two given tissues were compared with the chi-square test. Violin plots were created with the ggplot2 package for the R software [46]. Heat maps were drawn with the gplots package for the R software, using *Z*-score-normalized FA percent data [47].

## 3. Results

### 3.1. Effects of AA on Male Reproductive System Organ FA

A total of 24 FAs were detectable across tissues. All FA data are available in Table S2.

**Figure 1:**
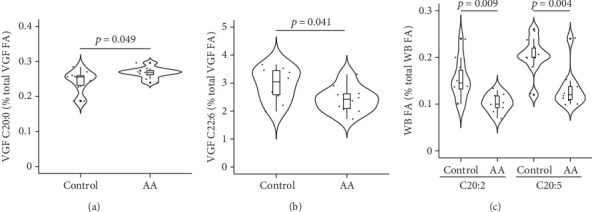
Significant effects of AA supplementation on FA in the male reproductive system and in whole blood. FA is significantly different between AA-supplemented and control mice in the VGF (a, b) or in the WB (c). VGF, vesicular gland fluid. WB, whole blood. *n* = 10/group. Mann–Whitney *U* test.

Among the male reproductive system–related fluids or tissues TIF, EAT, and VGF, only VGF showed a significant effect of supplementation with AA on FA profile: Arachidic acid (C20:0) was higher, and docosahexaenoic acid (C22:6) was lower in the AA-supplemented group compared with control mice (Figures 1(a) and 1(b)). Additionally, VGF palmitoleic acid (C16:1n-7) was lower in the AA-supplemented group relative to controls but at borderline significance (0.29% and 0.39%, respectively; *p* = 0.077). None of those FAs was affected by AA supplementation in WB, where eicosadienoic acid (C20:2) and eicosapentaenoic acid (C20:5) were significantly higher in the AA-supplemented group (Figure 1(c)). Conversely, no FA was affected by AA supplementation in the IBAT.

### 3.2. Laterality of TIF FA

**Figure 2:**
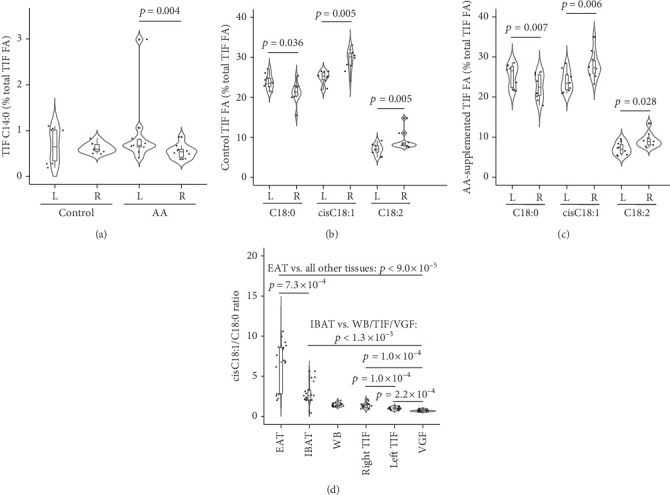
AA-associated and AA-independent laterality of FA in the testicular interstitial fluid. (a) AA-dependent effects on C14:0 in the right and left (R and L, respectively) TIF. (b, c) AA-independent significant differences in FA between the right and left TIF. (d) Oleic/stearic acid (cisC18:1/C18:0) ratio across tissues ranked left to right by decreasing values. Wilcoxon paired test. *n* = 10/group, except *n* = 9 in AA-supplemented IBAT. EAT, epididymal adipose tissue. IBAT, interscapular brown adipose tissue. TIF, testicular interstitial fluid. VGF, vesicular gland fluid. WB, whole blood.

Our study design allowed for the assessment of asymmetry in FA between left and right testis TIFs. We uncovered both AA-dependent and AA-independent lateralities of TIF FA. As for AA-dependent differences, myristic acid (C14:0) was significantly higher in the left compared to the right TIF, but only in the AA-supplemented group (Figure 2(a)). On the other hand, three FAs showed asymmetry between left and right TIFs from both AA-supplemented and control mice. These were stearic acid (C18:0) that was significantly higher in the left compared to right TIF and oleic (cisC18:1) and linoleic (C18:2) acid that changed in the opposite direction (Figures 2(b) and 2(c)). The data suggested a higher desaturation of C18:0 to cisC18:1 in the right compared to the left TIF, indicative of increased SCD1 activity [48]. Accordingly, the cisC18:1/C18:0 ratio was significantly higher in the right compared to left TIF, when compounded TIF from AA-supplemented and control TIF were compared (right TIF: 1.35 ± 0.32; left TIF: 1.00 ± 0.17; *p* = 1.0 × 10^−4^; Wilcoxon paired test). SCD1 can also desaturate palmitic acid to palmitoleic acid (C16:0 and C16:1n-7, respectively), yet their ratio was not significantly different between the left and right TIFs (0.04 ± 0.03 and 0.04 ± 0.02, respectively, *p* = 0.526; Wilcoxon paired test); that result could be explained by preferential elongation of C16:0 to C18:0 [49]. Incidentally, we observed a tendency for the right testis to be heavier than the left counterpart as previously reported, but the difference did not reach significance and AA supplementation had no effect (AA-supplemented and control testes compounded: right: 103.0 ± 20.0 mg; left: 95.9 ± 24.7 mg; *p* = 0.362; Mann–Whitney *U* test) [42]. Our study design also allowed for comparisons of SCD1 activity among tissues. The cisC18:1/C18:0 ratio was highly different among tissues (*p* < 1.0 × 10^−10^, Kruskal–Wallis ANOVA). The EAT showed markedly high values that were significantly different from all other tissues, although relatively more similar to the IBAT. The remaining tissues were relatively close although significantly different in most cases (Figure 2(d)).

### 3.3. Tissue Specificity of FA Profiles

**Figure 3:**
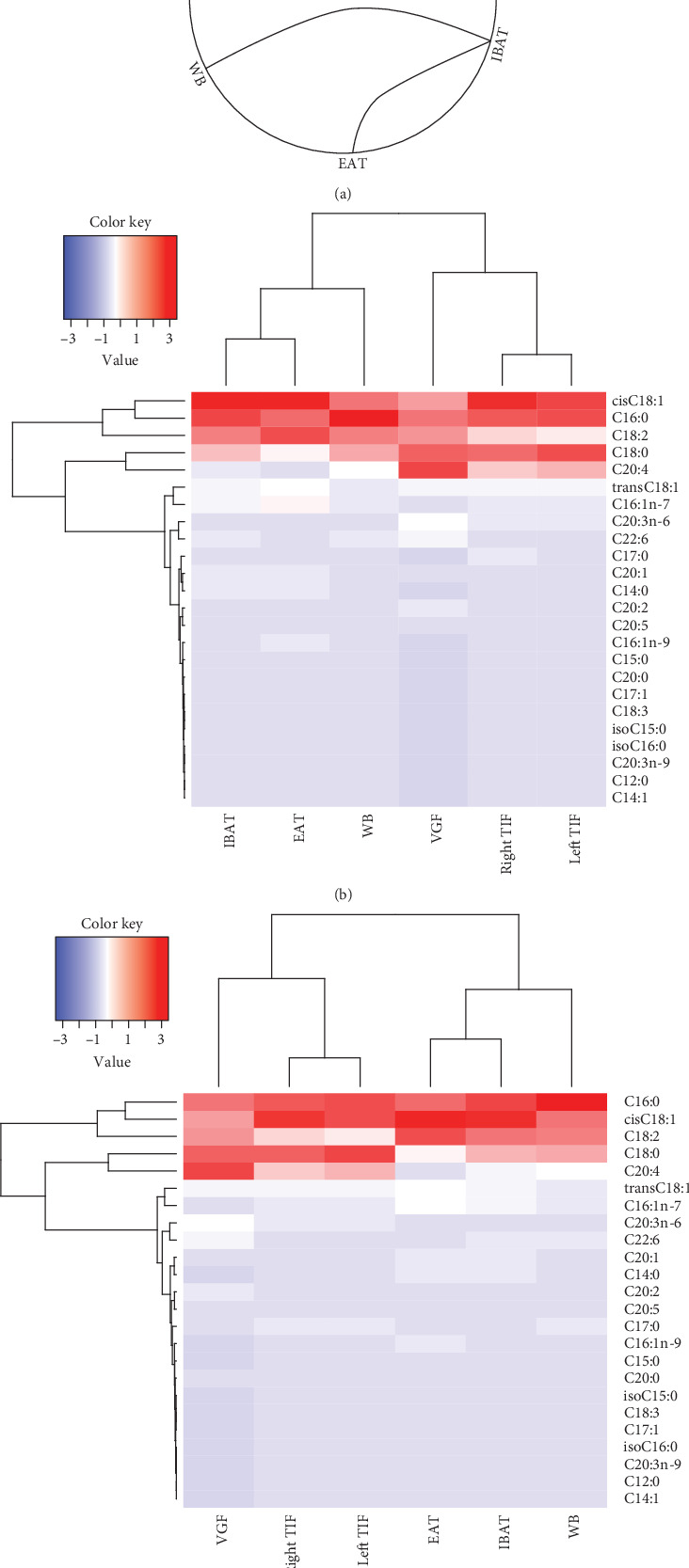
Multitissue comparison of FA profiles. (a) Circos graph of paired comparisons across tissues. Solid curved lines connect tissues with nonsignificantly different FA profiles (chi-square test). (b, c) Heat maps of individual FA levels (listed on the right-hand side of the heat map) across the analyzed tissues (listed below the heat map) in control (b) or AA-supplemented (c) mice. The left-to-right order of tissues in (b, c) is as rendered by the R software. *n* = 10/group, except *n* = 9 in AA-supplemented IBAT. EAT, epididymal adipose tissue. IBAT, interscapular brown adipose tissue. TIF, testicular interstitial fluid. VGF, vesicular gland fluid. WB, whole blood.

We expanded the comparison across tissues to assess whether FA profiles differed between the male reproductive system and the reference counterparts WB and IBAT. Paired comparisons of compounded AA-supplemented and control mouse FA identified tissue groups with nonsignificantly different FA profiles: one group was composed by the two TIFs and the VGF and the other group by the WB, EAT, and IBAT (Figure 3(a)). To identify individual FA that accounted for that grouping, we performed cluster analysis in the AA-supplemented and control group separately. The data revealed that abundant FAs such as cisC18:1, C16:0, C18:0, and C18:2 were highly variable across tissues while low-abundance FAs display relatively similar patterns and low variation among tissues (e.g., most long-chain PUFA and branched FA). We also found that FA abundance varied between the two tissue groups previously identified irrespective of AA supplementation (i.e., TIF and VGF on the one hand and WB, EAT, and IBAT on the other hand), particularly for AA (C20:4) (Figures 3(b) and 3(c)). Indeed, AA, in addition to C18:0 and C18:2, showed significant differences between the two tissue groups (*p* range: 4.3 × 10^−3^–1.2 × 10^−12^; Kruskal–Wallis ANOVA and Wilcoxon paired test).

Our FA determination method also detected the branched FA 14-methyl-C15:0 (isoC15:0) and 15-methyl-C16:0 (isoC16:0). Branched FAs may play a role in reproduction and in general physiology, as they are decreased in obese rodents and may have anti-inflammatory and anticancer activity [50–52]. Branched FAs were detected in the EAT and IBAT and at relatively low levels in the VGF and WB but were undetectable in TIF (Table S3). AA supplementation did not affect branched FA in any of the surveyed tissues.

## 4. Discussion

Our data confirmed the initial hypothesis that supplementation with AA modifies the FA content of the male reproductive system. Additionally, to our knowledge for the first time, we documented selected testis FA laterality that was both dependent and independent of AA supplementation. Multitissue analysis of FA profiles revealed two clusters independent of AA supplementation, one composed of the TIF and VGF and the other composed of the adipose tissue and WB.

Selected long-chain FAs (≥ 20 carbons) were altered by AA supplementation in the male reproductive system. The data highlighted notable features of the response to AA. Firstly, we observed a marked tissue specificity as only VGF FAs were affected by AA. Secondly, AA affected different long-chain FAs in the WB and in the VGF. The data indicate that the VGF responded to AA by modifying FA metabolism or uptake, rather than passively acquiring the WB FA profile. The data presented here converge with our previous studies of intergenerational effects of maternal and/or paternal AA supplementation. Notably, C20:0 (increased by AA in the VGF in this study) showed a cumulative change in livers across three consecutive offspring generations in a mouse model of paternal AA supplementation, although the association with the amount of supplemented AA was negative [10]. Likewise, C20:5 (decreased by AA in WB) was increased in livers in the same study. In a separate study of intergenerational effects of paternal AA supplementation, C20:5 content was higher in blood of AA-supplemented compared to vector control offspring [11]. The latter study also documented a decline in brain PUFA, which mirrors the decrease in C22:6 and C20:5 induced by AA in the VGF and WB, respectively, observed in the present study. Additionally, liver C16:1n-7 showed a borderline significant negative association with paternal AA [10] and was borderline significantly lower in the AA-supplemented VGF. Overall, our data confirm the preferential effect of AA on long-chain FA. The inconsistencies in the direction of associations likely reflects differences between supplemented founders and their progeny, and tissue-specific effects. Finally, it is noteworthy that the results of the mentioned studies generally converge with the present one, despite being conducted in different mouse strains—that is, BALB/c or C57BL/6, respectively [10, 11].

Our data suggest that VGF FA may participate in the intergenerational effects induced by AA. That model is supported by the previously documented participation of the seminal fluid in paternal intergenerational programming in rodents [18–21]. Considering that AA decreases VGF C22:6—an anti-inflammatory FA—we speculate that the exposure to AA blunts the inflammatory response of the uterine wall via the sperm, impacting gene expression in the oocyte, the zygote, and possibly the adult offspring [53, 54]. That view is supported by evidence that PPAR signalling mediates the transgenerational effects of the paternal exposure to traumatic stress in a rodent model and in humans [35]. Notably, PUFAs such as C22:6 are endogenous PPAR ligands [55, 56]. In fact, C20:5 (decreased by AA in the WB) was among the circulating factors specifically altered by paternal stress in mice [35]. PPAR signalling may participate in the paternal effects of a variety of exposures, and the relative contribution of pathways additional to PPAR signalling may be responsible for type of exposure-specific intergenerational effects. An additional participating pathway may be the sphingosine-1-phosphate receptor 1 signalling, which was recently shown to mediate the biological effects of C20:5 [57]. AA-modified FA profiles, particularly long-chain PUFA, are associated with epigenetic changes that may explain intergenerational effects. In experimental studies, consistent with the aforementioned data, in a cell culture model, AA-induced DNA hypermethylation was mediated by PPAR*α*, possibly secondary to the generation of PPAR ligands [34]. Also, C22:6 and C20:5 induced DNA hypermethylation in cell culture, although that response was cell line dependent [58]. As for human intervention studies, C22:6 supplementation was associated with gene-specific and global DNA methylation alterations [59]. A similar conclusion has been reached by n-3 PUFA supplementation [60–62]. Those data echo the observation that endogenous C20:5 was directly associated with global DNA methylation in humans [63]. This growing area of research has been reviewed with an emphasis on human reproduction and development [64, 65].

Our study identified a significant laterality in the testis. Myristic acid (C14:0) was increased by AA, but not the vehicle control, in the left relative to the right TIF. Notably, brain C14:0 was modulated by paternal AA in the unsupplemented progeny in both males and females [11]. Another observed testis laterality was the ratio between C18:0 and its desaturation products cisC18:1 and C18:2, suggestive of higher SCD1 activity in the right testis, irrespective of AA supplementation. Both cases of testis laterality may impose differential cellular phenotypes in the germline and in sperm cells. C14:0 diminishes inflammation and oxidative stress in the testis [66]. Regulation of C14:0 and C22:6 (see above) by AA may therefore fine-tune the inflammatory potential of the sperm. SCD1 activity shapes the cellular FA pool and may induce heritable epigenetic marks by interacting with the DNA methylation machinery [67, 68]. We acknowledge that the lack of additional data on SCD1 activity is a limitation of this study. Nonetheless, whether directly driven by SCD1, the excess of oleic relative to stearic acid is expected to exert an anti-inflammatory milieu in the right testis [69]. Therefore, testis laterality may modulate the intergenerational information and performance of the sperm cell, with possible consequences for the reproductive physiology and assisted reproduction.

Beyond the effects of AA, we documented differences in FA between the male reproductive system and nonreproductive tissues. FA profiles of the left and right TIF and the VGF were more similar than the EAT, IBAT, and WB counterparts. Highly abundant FAs, particularly tissue AA, were differentially present between those two clusters. The data indicate that FA fingerprints are broadly associated with tissue physiological functions, although our data cannot indicate any direction of causality.

The observed distribution of branched FA was in part expected, as they are endogenously synthesized in the adipose tissue [52]. To our knowledge, the presence of branched FA in the VGF has not been reported. Given that we detected branched FA in the WB, we cannot conclude whether the vesicular gland can synthesize branched FA or uptakes them from the circulating pool. The absence of branched FA in the TIF is noteworthy. A possible explanation is that excluding branched FA from the germline environment is evolutionarily advantageous. This speculative idea is supported by the documented proapoptotic activity of branched FA [70]. Further experimental work is warranted to assess the functional significance of the absence of branched FA in the TIF.

One weakness of our study is that we base our conclusions on nominal *p* values. Nonetheless, the manyfold coincidences between our data and the conclusions reached by other models of intergenerational transmission, particularly the sensitivity of long-chain PUFA to AA, assign biological significance to our findings.

## 5. Conclusion

We highlight AA supplementation–dependent and AA supplementation–independent FA profiles in the male reproductive system that may broaden our understanding of the molecular mechanisms of paternal inheritance and general aspects of male reproductive biology.

## Data Availability

The data that support the findings of this study are available in the supporting information of this article.

## Supporting Information

Supporting Information Additional supporting information can be found online in the Supporting Information section. Table S1: Description of the fatty acid profile of the vehicle (Nutrioli soybean oil). Table S2: Detected fatty acids (each expressed as percentage of total fatty acid) in the surveyed tissues. This table contains the original data. Table S3: Abundance of branched fatty acids across the surveyed tissues.
