# Supplementary material for: A Survey of Fatty Acid Content of the Male Reproductive System in Mice Supplemented With Arachidonic Acid
Source: J Lipids. 2024 Dec 19;2024:3351340. doi: 10.1155/jl/3351340 (PMC11671656; doi:10.1155/jl/3351340)
Supplement: Supporting Information — Additional supporting information can be found online in the Supporting Information section. Table S1: Description of the fatty acid profile of the vehicle (Nutrioli soybean oil). Table S2: Detected fatty acids (each expressed as percentage of total fatty acid) in the surveyed tissues. This table contains the original data. Table S3: Abundance of branched fatty acids across the surveyed tissues. [file 3351340.f1.zip › 3351340_Supplemental_Tables.pdf]

Supplementary material

Manuscript title: A survey of fatty acid content of the male reproductive system in mice supplemented with arachidonic acid

First author's surname: Correa-Navarro

Content:

Supplementary Table 1

Supplementary Table 2

Supplementary Table 3

Supplementary Table 1. Fatty acid profile of the vehicle (Nutrioli® soybean oil).

Fatty acid	% of total fatty acids
C14:0 (Myristic)	ND
C16:0 (Palmitic)	10.85
C18:0 (Stearic)	4.76
C20:0 (Arachidic)	ND
C16:1n-9 (cis-7-hexadecenoic)	ND
C16:1n-7 (Palmitoleic)	ND
C18:1c (Oleic)	20.92
C18:1t (Elaidic)	2.35
C20:1 (Eicosenoic)	ND
C18:2n-6 (Linoleic)	54.31
C20:4n-6 (Arachidonic)	ND
C:20:3n-6 (DGLA)	5.38
C18:3n-3 (alpha-linolenic)	ND
C20:5n-3 (EPA)	ND
C20:6n-3 (DHA)	ND

ND, not detected. DGLA, dihomo-gamma-linoleic acid.
EPA, eicosapentaenoic acid. DHA, docosahexaenoic acid.

Supplementary Table 2. Detected fatty acids (each expressed as % of total fatty acid) in the surveyed tissues.

EAT	Mouse ID	1	2	3	4	5	6	7	8	9	10	11	12	13	14	15	16	17	18	19	20
	Group	AA	AA	AA	AA	AA	AA	AA	AA	AA	AA	Control	Control	Control	Control	Control	Control	Control	Control	Control	Control
Fatty acid																					
C12:0		0.03	0.03	0.04	0.01	0.02	0.03	0.01	0.04	0.03	0.01	0.01	0.03	0.04	0.01	0.02	0.03	0.02	0.04	0.02	0.05
C14:1		0.04	0.01	0.01	0.01	0.03	0.02	0.01	0.02	0.01	0.01	0.02	0.03	0.04	0.01	0.03	0.02	0.01	0.02	0.01	0.01
C14:0		0.99	1.35	1.38	0.75	0.97	1.09	0.71	1.41	1.30	0.78	0.82	1.03	1.62	0.88	1.03	1.34	0.88	1.54	0.74	1.59
C15:0		0.22	0.24	0.26	0.17	0.20	0.22	0.17	0.24	0.26	0.18	0.15	0.22	0.25	0.19	0.21	0.26	0.16	0.30	0.16	0.27
isoC15:0		0.14	0.21	0.23	0.10	0.14	0.15	0.09	0.20	0.19	0.10	0.11	0.16	0.22	0.11	0.17	0.22	0.10	0.22	0.08	0.22
C16:1-9		0.53	0.70	0.62	0.38	0.54	0.62	0.53	0.70	0.67	0.53	0.51	0.58	0.69	0.58	0.53	0.67	0.54	0.79	0.51	0.75
C16:1n-7		7.82	3.90	3.64	4.66	6.69	5.63	4.82	3.40	3.50	4.75	4.94	7.17	5.87	5.56	7.38	5.25	7.04	4.26	4.92	3.60
C16:0		21.72	24.56	24.28	20.08	20.17	22.28	20.01	25.06	22.66	22.40	20.66	20.41	23.97	22.13	21.60	22.08	19.34	23.97	20.12	24.56
isoC16:0		0.03	0.06	0.06	0.03	0.03	0.04	0.03	0.06	0.06	0.03	0.03	0.04	0.06	0.03	0.04	0.06	0.03	0.06	0.03	0.07
C17:1		0.38	0.36	0.29	0.24	0.35	0.35	0.26	0.31	0.32	0.28	0.23	0.40	0.34	0.30	0.36	0.35	0.32	0.37	0.19	0.31
C17:0		0.26	0.47	0.52	0.28	0.27	0.33	0.27	0.50	0.54	0.31	0.25	0.32	0.39	0.29	0.26	0.42	0.24	0.56	0.25	0.55
C18:3		0.28	0.04	0.07	0.34	0.32	0.54	0.44	0.02	0.06	0.45	0.30	0.22	0.03	0.22	0.31	0.11	0.48	0.03	0.23	0.18
C18:2		24.56	24.63	24.76	25.04	25.23	25.29	25.25	25.43	25.33	24.37	24.40	26.07	25.65	23.86	25.52	26.26	25.02	27.21	22.22	23.78
cisC18:1		34.37	25.55	26.37	38.96	35.58	32.44	37.57	24.82	27.30	35.51	38.74	32.28	24.34	34.92	33.37	28.64	36.71	21.79	42.53	22.47
transC18:1		3.11	4.63	4.17	2.81	3.38	3.43	2.63	4.38	4.38	3.33	2.93	3.82	4.92	3.66	3.29	4.27	3.37	5.17	2.79	5.14
C18:0		3.45	9.25	9.37	4.50	4.23	5.26	4.92	9.63	9.47	5.09	4.39	4.69	7.49	5.22	3.89	6.76	3.98	9.76	4.01	11.06
C20:4		0.49	0.52	0.47	0.25	0.34	0.41	0.98	0.44	0.43	0.32	0.24	0.53	0.43	0.35	0.31	0.41	0.39	0.49	0.16	0.50
C20:5		0.04	0.07	0.06	0.01	0.03	0.04	0.00	0.04	0.05	0.02	0.01	0.09	0.06	0.03	0.07	0.05	0.03	0.07	0.00	0.09
C20:3n-9		0.06	0.07	0.06	0.03	0.04	0.05	0.03	0.06	0.05	0.04	0.02	0.06	0.07	0.05	0.05	0.05	0.05	0.06	0.02	0.07
C20:3n-6		0.15	0.23	0.19	0.12	0.13	0.19	0.14	0.22	0.21	0.14	0.11	0.19	0.21	0.16	0.15	0.19	0.16	0.24	0.10	0.25
C20:2		0.33	0.65	0.61	0.31	0.36	0.38	0.23	0.66	0.62	0.33	0.29	0.42	0.65	0.37	0.36	0.54	0.31	0.70	0.23	0.79
C20:1		0.68	1.76	1.84	0.67	0.68	0.83	0.60	1.68	1.90	0.76	0.66	0.80	1.96	0.77	0.77	1.50	0.55	1.60	0.59	2.69
C20:0		0.06	0.23	0.21	0.07	0.07	0.10	0.06	0.22	0.22	0.08	0.08	0.08	0.22	0.10	0.08	0.15	0.07	0.18	0.06	0.30
C22:6		0.24	0.47	0.50	0.18	0.19	0.28	0.23	0.45	0.45	0.16	0.08	0.37	0.48	0.20	0.22	0.37	0.20	0.57	0.04	0.71

IBAT	Mouse ID	1	2	3	4	5	6	7	8	9	10	11	12	13	14	15	16	17	18	19	20
	Group	AA	AA	AA	AA	AA	AA	AA	AA	AA	AA	Control	Control	Control	Control	Control	Control	Control	Control	Control	Control
Fatty acid																					
C12:0		0.00	0.04	0.04	0.03	0.00	0.03	n.d.	0.00	0.04	0.04	0.03	0.02	0.05	0.00	0.00	0.00	0.00	0.03	0.00	0.03
C14:1		0.00	0.01	0.01	0.00	0.00	0.00	n.d.	0.00	0.01	0.01	0.00	0.00	0.03	0.00	0.00	0.00	0.00	0.00	0.00	0.01
C14:0		0.49	1.59	1.40	1.23	0.47	0.94	n.d.	0.00	1.51	1.60	1.36	1.11	2.02	0.00	0.00	0.73	0.67	1.22	0.82	1.54
C15:0		0.12	0.19	0.18	0.17	0.00	0.12	n.d.	0.00	0.24	0.22	0.10	0.17	0.08	0.00	0.00	0.13	0.06	0.18	0.14	0.17
isoC15:0		0.08	0.21	0.19	0.15	0.00	0.11	n.d.	0.00	0.19	0.20	0.09	0.17	0.07	0.00	0.00	0.23	0.13	0.27	0.23	0.18
C16:1-9		0.22	0.50	0.43	0.42	0.17	0.40	n.d.	0.00	0.58	0.55	0.39	0.45	0.30	0.00	0.17	0.28	0.31	0.44	0.30	0.45
C16:1n-7		1.52	2.39	2.23	1.55	1.94	1.70	n.d.	0.44	2.39	2.61	1.72	1.48	3.27	0.55	3.17	4.09	1.89	1.83	3.38	2.43
C16:0		29.49	24.65	24.25	20.47	30.38	20.21	n.d.	35.19	23.61	23.29	23.56	20.67	27.12	34.72	30.85	25.51	24.91	23.35	25.21	23.07
isoC16:0		0.04	0.05	0.05	0.04	0.00	0.04	n.d.	0.00	0.06	0.05	0.03	0.03	0.03	0.00	0.00	0.06	0.03	0.04	0.04	0.05
C17:1		0.03	0.08	0.15	0.16	0.00	0.13	n.d.	0.00	0.20	0.22	0.14	0.14	0.06	0.98	0.00	0.08	0.22	0.15	0.03	0.16
C17:0		0.26	0.44	0.43	0.41	0.25	0.29	n.d.	0.00	0.54	0.50	0.28	0.43	0.22	0.35	0.31	0.17	0.29	0.37	0.25	0.38
C18:3		0.02	0.20	0.20	0.38	0.00	0.29	n.d.	0.00	0.18	0.16	0.34	0.24	0.18	0.00	0.00	0.05	0.05	0.33	0.09	0.55
C18:2		16.37	20.97	22.22	25.02	15.79	23.68	n.d.	8.67	23.74	23.71	21.66	24.46	18.69	9.10	15.92	18.53	19.27	23.87	20.92	22.54
cisC18:1		28.26	28.45	29.18	32.53	28.96	38.63	n.d.	18.08	25.73	25.73	34.30	32.07	30.60	9.63	28.96	36.60	37.87	32.81	37.44	28.97
transC18:1		2.37	3.40	3.04	2.66	2.18	2.43	n.d.	2.60	3.74	3.96	2.19	3.13	2.01	2.40	2.53	2.65	2.54	2.53	2.51	2.89
C18:0		14.07	12.82	11.27	11.31	14.57	7.97	n.d.	26.57	12.31	12.55	10.95	11.88	11.71	22.52	13.33	7.49	8.66	9.93	6.65	12.16
C20:4		4.53	0.48	0.64	0.46	1.87	0.41	n.d.	8.45	0.54	0.46	0.43	0.39	0.45	6.37	1.90	1.29	0.95	0.43	0.65	0.59
C20:5		0.00	0.07	0.06	0.03	0.00	0.00	n.d.	0.00	0.07	0.06	0.02	0.02	0.04	0.00	0.00	0.00	0.00	0.04	0.00	0.09
C20:3n-9		0.00	0.02	0.03	0.01	0.00	0.02	n.d.	0.00	0.04	0.05	0.02	0.02	0.02	0.00	0.00	0.00	0.00	0.02	0.00	0.04
C20:3n-6		0.12	0.16	0.13	0.12	0.00	0.11	n.d.	0.00	0.16	0.15	0.10	0.13	0.10	0.00	0.00	0.05	0.05	0.11	0.04	0.16
C20:2		0.21	0.51	0.53	0.42	0.00	0.36	n.d.	0.00	0.62	0.60	0.33	0.52	0.31	0.00	0.00	0.13	0.13	0.41	0.13	0.48
C20:1		0.40	2.02	2.33	1.71	0.14	1.52	n.d.	0.00	2.62	2.48	1.34	1.84	1.70	0.00	0.00	0.54	0.37	1.17	0.56	2.17
C20:0		0.06	0.44	0.39	0.27	0.00	0.24	n.d.	0.00	0.41	0.39	0.28	0.32	0.69	0.00	0.00	0.08	0.00	0.19	0.00	0.30
C22:6		1.32	0.30	0.63	0.43	3.27	0.37	n.d.	0.00	0.49	0.41	0.34	0.29	0.26	13.37	2.86	1.33	1.59	0.27	0.62	0.62

Left TIF	Mouse ID	1	2	3	4	5	6	7	8	9	10	11	12	13	14	15	16	17	18	19	20
	Group	AA	AA	AA	AA	AA	AA	AA	AA	AA	AA	Control	Control	Control	Control	Control	Control	Control	Control	Control	Control
Fatty acid																					
C12:0		0.00	0.00	0.00	0.00	0.00	0.00	0.00	0.00	0.00	0.00	0.00	0.00	0.00	0.00	0.00	0.00	0.00	0.00	0.00	0.00
C14:1		0.00	0.00	0.00	0.00	0.00	0.00	0.00	0.00	0.00	0.00	0.00	0.00	0.00	0.00	0.00	0.00	0.00	0.00	0.00	0.00
C14:0		1.06	0.66	0.67	0.53	0.83	0.72	0.41	0.68	0.64	2.99	1.10	1.04	1.01	0.28	0.52	0.54	1.01	0.28	0.75	0.19
C15:0		0.50	0.36	0.39	0.28	0.30	0.27	0.22	0.37	0.84	0.39	0.77	0.87	0.21	0.10	0.17	0.31	0.20	0.40	0.15	0.04
isoC15:0		0.00	0.00	0.00	0.00	0.00	0.00	0.00	0.00	0.00	0.00	0.00	0.00	0.00	0.00	0.00	0.00	0.00	0.00	0.00	0.00
C16:1-9		0.00	0.00	0.00	0.00	0.00	0.00	0.00	0.00	0.00	0.00	0.00	0.00	0.00	0.00	0.00	0.00	0.00	0.00	0.00	0.00
C16:1n-7		1.91	1.38	0.00	0.69	0.81	0.57	0.32	0.60	0.57	1.03	0.57	2.93	2.00	0.86	0.87	0.54	0.78	1.81	0.64	0.47
C16:0		25.80	23.05	23.35	21.56	25.50	24.28	22.84	26.79	21.80	23.14	25.98	26.95	22.62	24.83	22.43	28.00	24.69	24.66	21.96	21.54
isoC16:0		0.00	0.00	0.00	0.00	0.00	0.00	0.00	0.00	0.00	0.00	0.00	0.00	0.00	0.00	0.00	0.00	0.00	0.00	0.00	0.00
C17:1		0.00	0.00	0.00	0.00	0.00	0.00	0.00	0.00	0.00	0.00	0.00	0.00	0.00	0.00	0.00	0.00	0.00	0.00	0.00	0.00
C17:0		1.43	1.19	0.62	0.67	1.25	0.62	0.75	0.90	0.74	0.87	0.85	0.75	0.59	0.71	0.53	0.79	0.51	0.77	0.54	0.69
C18:3		0.00	0.00	0.00	0.00	0.00	0.00	0.00	0.00	0.00	0.00	0.00	0.00	0.00	0.00	0.00	0.00	0.00	0.00	0.00	0.00
C18:2		5.49	5.30	5.60	8.37	8.95	7.26	6.73	6.56	7.42	9.54	8.23	6.93	6.22	9.18	7.04	5.02	7.96	5.16	7.58	6.54
cisC18:1		21.68	21.17	20.93	23.55	27.26	22.25	23.27	25.66	25.12	28.42	25.29	22.20	21.92	26.53	25.06	25.36	26.52	24.22	25.45	26.40
transC18:1		1.39	2.68	2.12	3.02	2.44	1.92	2.98	2.73	2.52	2.66	2.24	1.77	3.35	3.11	3.18	2.30	2.57	1.95	2.33	2.84
C18:0		28.55	27.84	27.96	23.64	21.58	26.85	26.16	21.92	27.13	21.65	21.51	22.68	22.86	23.65	23.35	24.38	21.30	24.87	26.11	27.16
C20:4		10.17	14.93	17.12	16.28	9.55	13.94	14.78	12.16	12.11	8.11	12.29	12.49	17.85	8.97	15.50	10.30	12.46	14.06	13.14	12.04
C20:5		0.00	0.00	0.00	0.00	0.00	0.00	0.00	0.00	0.00	0.00	0.00	0.00	0.00	0.00	0.00	0.00	0.00	0.00	0.00	0.00
C20:3n-9		0.00	0.00	0.00	0.00	0.00	0.00	0.00	0.00	0.00	0.00	0.00	0.00	0.00	0.00	0.00	0.00	0.00	0.00	0.00	0.00
C20:3n-6		0.51	0.87	1.04	0.74	0.81	0.99	0.95	1.17	0.73	0.65	0.80	0.85	0.91	1.01	0.85	0.55	1.38	0.87	1.01	1.16
C20:2		0.00	0.00	0.00	0.00	0.00	0.00	0.00	0.00	0.00	0.00	0.00	0.00	0.00	0.00	0.00	0.00	0.00	0.00	0.00	0.00
C20:1		1.51	0.59	0.19	0.66	0.73	0.34	0.59	0.46	0.36	0.56	0.38	0.55	0.45	0.78	0.52	1.92	0.63	0.96	0.35	0.91
C20:0		0.00	0.00	0.00	0.00	0.00	0.00	0.00	0.00	0.00	0.00	0.00	0.00	0.00	0.00	0.00	0.00	0.00	0.00	0.00	0.00
C22:6		0.00	0.00	0.00	0.00	0.00	0.00	0.00	0.00	0.00	0.00	0.00	0.00	0.00	0.00	0.00	0.00	0.00	0.00	0.00	0.00

Rught TIF	Mouse ID	1	2	3	4	5	6	7	8	9	10	11	12	13	14	15	16	17	18	19	20
	Group	AA	AA	AA	AA	AA	AA	AA	AA	AA	AA	Control	Control	Control	Control	Control	Control	Control	Control	Control	Control
Fatty acid																					
C12:0		0.00	0.00	0.00	0.00	0.00	0.00	0.00	0.00	0.00	0.00	0.00	0.00	0.00	0.00	0.00	0.00	0.00	0.00	0.00	0.00
C14:1		0.00	0.00	0.00	0.00	0.00	0.00	0.00	0.00	0.00	0.00	0.00	0.00	0.00	0.00	0.00	0.00	0.00	0.00	0.00	0.00
C14:0		0.49	0.57	0.51	0.39	0.37	0.59	0.37	0.67	0.56	0.86	0.50	0.49	0.72	0.66	0.54	0.71	0.83	0.58	0.61	0.58
C15:0		0.67	0.15	0.16	0.11	0.07	0.34	0.15	0.43	0.40	0.65	0.23	0.14	0.71	0.28	0.29	0.33	0.68	0.36	0.29	0.35
isoC15:0		0.00	0.00	0.00	0.00	0.00	0.00	0.00	0.00	0.00	0.00	0.00	0.00	0.00	0.00	0.00	0.00	0.00	0.00	0.00	0.00
C16:1-9		0.00	0.00	0.00	0.00	0.00	0.00	0.00	0.00	0.00	0.00	0.00	0.00	0.00	0.00	0.00	0.00	0.00	0.00	0.00	0.00
C16:1n-7		0.74	0.70	0.55	1.07	0.71	1.56	0.64	0.87	0.39	2.05	0.70	0.72	1.00	1.10	1.41	0.64	1.10	1.28	0.58	1.12
C16:0		27.73	24.60	22.73	20.70	20.47	28.18	21.91	25.21	21.03	24.84	21.29	21.71	26.85	27.20	19.17	20.55	29.63	22.34	23.06	26.70
isoC16:0		0.00	0.00	0.00	0.00	0.00	0.00	0.00	0.00	0.00	0.00	0.00	0.00	0.00	0.00	0.00	0.00	0.00	0.00	0.00	0.00
C17:1		0.00	0.00	0.00	0.00	0.00	0.00	0.00	0.00	0.00	0.00	0.00	0.00	0.00	0.00	0.00	0.00	0.00	0.00	0.00	0.00
C17:0		1.34	0.46	0.70	1.00	0.92	1.17	0.59	0.71	0.66	0.74	0.50	0.54	0.90	0.98	0.40	0.67	1.19	0.80	1.07	0.86
C18:3		0.00	0.00	0.00	0.00	0.00	0.00	0.00	0.00	0.00	0.00	0.00	0.00	0.00	0.00	0.00	0.00	0.00	0.00	0.00	0.00
C18:2		8.09	7.68	7.32	9.78	13.43	8.05	8.92	9.11	8.81	10.76	7.89	8.23	8.72	8.45	14.81	7.64	7.69	8.34	11.05	8.00
cisC18:1		27.07	25.62	23.26	27.51	31.55	28.06	25.24	29.32	26.88	35.02	26.52	24.05	30.47	31.96	33.04	27.95	31.17	28.18	30.76	29.86
transC18:1		1.74	2.27	2.23	3.44	2.57	1.80	2.26	2.05	2.53	2.02	2.68	1.94	2.30	2.17	2.96	3.13	1.74	2.11	2.28	2.42
C18:0		25.62	26.09	25.26	20.97	20.22	19.19	26.30	20.89	23.98	17.90	21.28	25.40	20.19	21.43	15.50	22.78	20.88	22.84	20.01	21.37
C20:4		5.85	10.94	16.04	13.68	8.29	10.12	12.43	9.44	13.38	4.30	15.91	15.24	7.50	3.73	10.59	13.82	4.15	11.68	9.27	7.88
C20:5		0.00	0.00	0.00	0.00	0.00	0.00	0.00	0.00	0.00	0.00	0.00	0.00	0.00	0.00	0.00	0.00	0.00	0.00	0.00	0.00
C20:3n-9		0.00	0.00	0.00	0.00	0.00	0.00	0.00	0.00	0.00	0.00	0.00	0.00	0.00	0.00	0.00	0.00	0.00	0.00	0.00	0.00
C20:3n-6		0.24	0.57	0.95	1.02	0.87	0.58	0.81	0.76	0.77	0.23	1.22	1.05	0.27	1.35	0.67	1.04	0.39	0.86	0.59	0.55
C20:2		0.00	0.00	0.00	0.00	0.00	0.00	0.00	0.00	0.00	0.00	0.00	0.00	0.00	0.00	0.00	0.00	0.00	0.00	0.00	0.00
C20:1		0.42	0.34	0.28	0.31	0.52	0.37	0.39	0.52	0.60	0.65	1.27	0.51	0.37	0.69	0.63	0.74	0.55	0.62	0.43	0.32
C20:0		0.00	0.00	0.00	0.00	0.00	0.00	0.00	0.00	0.00	0.00	0.00	0.00	0.00	0.00	0.00	0.00	0.00	0.00	0.00	0.00
C22:6		0.00	0.00	0.00	0.00	0.00	0.00	0.00	0.00	0.00	0.00	0.00	0.00	0.00	0.00	0.00	0.00	0.00	0.00	0.00	0.00

VGF	Mouse ID	1	2	3	4	5	6	7	8	9	10	11	12	13	14	15	16	17	18	19	20
	Group	AA	AA	AA	AA	AA	AA	AA	AA	AA	AA	Control	Control	Control	Control	Control	Control	Control	Control	Control	Control
Fatty acid																					
C12:0		0.00	0.00	0.00	0.00	0.00	0.00	0.00	0.00	0.00	0.00	0.00	0.00	0.00	0.00	0.00	0.00	0.00	0.00	0.00	0.00
C14:1		0.00	0.00	0.00	0.00	0.00	0.00	0.00	0.00	0.00	0.00	0.00	0.00	0.00	0.00	0.00	0.00	0.00	0.00	0.00	0.00

C14:0		0.18	0.18	0.34	0.11	0.27	0.18	0.17	0.27	0.26	0.32	0.17	0.20	0.40	0.14	0.16	0.15	0.12	0.16	0.13	0.28
C15:0		0.06	0.09	0.04	0.06	0.03	0.06	0.07	0.05	0.04	0.07	0.08	0.07	0.09	0.07	0.10	0.08	0.08	0.05	0.04	0.05
isoC15:0		0.20	0.03	0.00	0.02	0.02	0.13	0.02	0.03	0.05	0.00	0.02	0.05	0.04	0.02	0.01	0.02	0.02	0.03	0.04	0.02
C16:1-9		0.00	0.00	0.00	0.00	0.00	0.00	0.00	0.00	0.00	0.00	0.00	0.00	0.00	0.00	0.00	0.00	0.00	0.00	0.00	0.00
C16:1n-7		0.60	0.21	0.21	0.18	0.22	0.41	0.35	0.25	0.32	0.22	0.27	0.52	0.50	0.18	0.34	0.41	0.41	0.50	0.50	0.30
C16:0		16.60	16.49	18.22	16.19	16.27	17.42	17.28	17.22	17.76	17.44	17.06	18.14	17.68	17.03	16.77	18.13	16.33	16.88	16.95	17.63
isoC16:0		0.00	0.00	0.00	0.00	0.00	0.00	0.00	0.00	0.00	0.00	0.00	0.00	0.00	0.00	0.00	0.00	0.00	0.00	0.00	0.00
C17:1		0.00	0.00	0.00	0.00	0.00	0.00	0.00	0.00	0.00	0.00	0.00	0.00	0.00	0.00	0.00	0.00	0.00	0.00	0.00	0.00
C17:0		0.25	0.29	0.21	0.38	0.25	0.26	0.24	0.22	0.35	0.19	0.23	0.21	0.24	0.19	0.24	0.28	0.22	0.30	0.34	0.39
C18:3		0.00	0.00	0.00	0.00	0.00	0.00	0.00	0.00	0.02	0.00	0.00	0.00	0.00	0.00	0.00	0.00	0.00	0.00	0.00	0.00
C18:2		15.12	14.96	14.94	13.45	15.58	14.06	12.39	15.04	14.06	13.34	15.32	15.19	14.26	12.78	15.76	13.49	15.54	14.25	13.45	13.21
cisC18:1		17.36	12.40	13.53	11.14	13.85	15.31	12.86	13.44	15.77	11.84	14.23	17.07	14.46	11.20	12.19	12.27	12.44	14.39	13.33	12.06
transC18:1		2.93	2.91	2.58	2.71	3.01	2.94	2.96	2.97	3.06	2.83	2.93	2.60	3.04	2.81	3.10	3.05	2.95	2.88	2.98	2.76
C18:0		18.33	19.47	19.78	20.18	19.60	18.54	18.96	18.85	17.30	20.48	18.42	17.50	20.78	19.33	19.27	18.22	19.68	19.18	18.66	18.93
C20:4		20.52	23.10	21.92	24.96	21.16	22.05	23.80	22.45	20.18	23.92	20.52	20.28	19.47	25.29	20.28	23.17	22.04	20.96	22.70	24.34
C20:5		0.56	1.08	0.75	0.99	0.94	0.66	0.84	0.69	0.76	0.82	1.11	0.66	0.55	0.87	1.27	0.88	0.90	0.71	0.70	0.65
C20:3n-9		0.00	0.00	0.00	0.00	0.00	0.00	0.00	0.00	0.00	0.00	0.00	0.00	0.00	0.00	0.00	0.00	0.00	0.00	0.00	0.00
C20:3n-6		3.19	4.07	3.66	5.37	4.22	3.36	5.39	3.60	4.26	3.32	4.46	3.55	3.78	4.26	5.05	4.63	3.81	4.13	4.60	3.45
C20:2		1.12	1.19	1.17	1.40	1.36	1.21	1.12	1.31	1.32	1.24	1.44	0.94	1.32	1.23	1.71	1.17	1.48	1.19	1.24	1.37
C20:1		0.71	0.80	0.67	0.53	0.78	0.78	0.65	0.78	0.95	0.75	0.94	0.61	1.12	0.68	0.83	0.69	0.78	0.67	0.72	0.76
C20:0		0.27	0.26	0.27	0.28	0.27	0.26	0.27	0.25	0.24	0.30	0.26	0.19	0.27	0.24	0.26	0.19	0.28	0.25	0.23	0.26
C22:6		1.99	2.48	1.72	2.06	2.17	2.37	2.63	2.58	3.32	2.91	2.55	2.21	1.99	3.66	2.69	3.18	2.91	3.46	3.38	3.53

**WB**	Mouse ID	1	2	3	4	5	6	7	8	9	10	11	12	13	14	15	16	17	18	19	20
	Group	AA	AA	AA	AA	AA	AA	AA	AA	AA	AA	Control	Control	Control	Control	Control	Control	Control	Control	Control	Control
Fatty acid																					
C12:0		0.00	0.00	0.00	0.00	0.00	0.00	0.00	0.00	0.00	0.00	0.00	0.00	0.00	0.00	0.00	0.00	0.00	0.00	0.00	0.00
C14:1		0.00	0.00	0.00	0.00	0.00	0.00	0.00	0.00	0.00	0.00	0.00	0.00	0.00	0.00	0.00	0.00	0.00	0.00	0.00	0.00
C14:0		0.08	0.13	0.11	0.19	0.10	0.22	0.33	0.09	0.12	0.13	0.17	0.10	0.14	0.17	0.14	0.15	0.12	0.22	0.07	0.19
C15:0		0.08	0.12	0.09	0.18	0.11	0.06	0.17	0.08	0.16	0.13	0.16	0.14	0.21	0.13	0.18	0.25	0.04	0.16	0.14	0.16
isoC15:0		0.05	0.04	0.05	0.05	0.02	0.03	0.04	0.02	0.03	0.02	0.00	0.00	0.05	0.00	0.06	0.08	0.03	0.03	0.02	0.02
C16:1-9		0.09	0.11	0.00	0.09	0.08	0.12	0.11	0.00	0.20	0.20	0.00	0.00	0.00	0.00	0.14	0.00	0.12	0.10	0.12	0.00
C16:1n-7		0.95	0.70	0.63	0.72	0.57	0.43	0.58	0.39	0.89	0.93	0.44	0.43	0.99	0.52	0.66	0.72	0.48	0.65	0.47	0.74
C16:0		38.43	33.71	34.75	30.31	36.77	34.27	36.77	35.96	34.55	34.62	36.92	35.10	32.82	34.34	31.65	32.74	36.89	35.67	35.49	35.17
isoC16:0		0.00	0.00	0.00	0.00	0.00	0.00	0.00	0.00	0.00	0.00	0.00	0.00	0.00	0.00	0.00	0.00	0.00	0.00	0.00	0.00
C17:1		0.00	0.00	0.00	0.00	0.00	0.00	0.00	0.00	0.00	0.00	0.00	0.00	0.00	0.00	0.00	0.00	0.00	0.00	0.00	0.00
C17:0		0.48	0.39	0.45	0.68	0.47	0.45	0.58	0.35	0.45	0.45	0.44	0.44	0.55	0.50	0.57	0.61	0.48	0.50	0.51	0.41
C18:3		0.00	0.00	0.00	0.06	0.00	0.00	0.00	0.00	0.00	0.00	0.00	0.00	0.10	0.00	0.04	0.07	0.00	0.00	0.00	0.00
C18:2		18.96	19.48	19.51	19.71	17.00	20.71	19.18	18.53	21.00	19.47	18.40	19.65	18.04	19.83	20.60	20.70	16.63	20.09	18.34	20.27
cisC18:1		19.14	20.81	21.33	24.40	19.99	19.10	19.56	18.34	21.22	20.56	18.62	18.51	26.10	19.57	23.73	23.11	20.87	18.61	19.55	20.26
transC18:1		1.95	1.67	1.43	1.27	1.73	1.52	1.63	1.30	1.50	1.60	1.44	1.40	1.85	1.53	1.86	1.75	1.56	1.49	1.76	1.65
C18:0		13.99	14.89	13.67	16.15	14.92	15.57	15.14	15.07	12.93	13.55	15.39	13.83	13.15	14.11	14.24	13.19	13.84	14.56	15.67	13.56
C20:4		3.91	5.73	5.56	4.08	5.95	5.35	4.10	7.41	4.77	5.89	6.06	7.57	3.47	6.52	3.65	4.20	5.96	5.39	5.33	5.06
C20:5		0.11	0.12	0.10	0.24	0.12	0.11	0.10	0.15	0.13	0.14	0.00	0.20	0.26	0.18	0.24	0.22	0.20	0.21	0.12	0.20
C20:3n-9		0.00	0.00	0.00	0.00	0.00	0.00	0.00	0.00	0.00	0.00	0.00	0.00	0.00	0.00	0.00	0.00	0.00	0.00	0.00	0.00
C20:3n-6		0.33	0.46	0.50	0.27	0.42	0.39	0.35	0.45	0.39	0.54	0.36	0.50	0.26	0.52	0.26	0.26	0.45	0.46	0.51	0.45
C20:2		0.10	0.10	0.09	0.12	0.12	0.11	0.07	0.10	0.08	0.13	0.13	0.10	0.14	0.10	0.15	0.20	0.18	0.15	0.24	0.14
C20:1		0.47	0.28	0.39	0.43	0.30	0.35	0.36	0.18	0.42	0.38	0.28	0.22	0.66	0.33	0.57	0.49	0.37	0.28	0.35	0.46
C20:0		0.24	0.28	0.29	0.29	0.23	0.30	0.32	0.16	0.29	0.20	0.20	0.19	0.28	0.23	0.32	0.26	0.22	0.27	0.23	0.30
C22:6		0.64	0.97	1.06	0.75	1.08	0.92	0.61	1.41	0.87	1.05	1.00	1.62	0.92	1.40	0.96	1.01	1.58	1.14	1.08	0.96

EAT, epididymal adipose tissue. IBAT, interscapular brown adipose tissue. TIF, testicular interstitial fluid. VGF, vesicular gland fluid. WB, whole blood.
AA, arachidonic acid-supplemented
n.d., not determined.

Supplementary Table 3. Abundance of branched fatty acids across the surveyed tissues.

	isoC15:0 (%)		isoC16:0 (%)	
Tissue	Control	AA	Control	AA
Left TIF	n.d.	n.d.	n.d.	n.d.
Right TIF	n.d.	n.d.	n.d.	n.d.
VGF	0.02±0.01	0.05±0.07	n.d.	n.d.
EAT	0.16±0.05	0.15±0.05	0.04±0.01	0.04±0.01
IBAT	0.14±0.07	0.12±0.07	0.03±0.02	0.04±0.02
WB	0.03±0.01	0.03±0.02	n.d.	n.d.

n=10/group, except n=9 in AA-supplemented IBAT.
EAT, epidydimal adipose tissue. IBAT, interscapular brown adipose tissue. TIF, testicular interstitial fluid. VGF, vesicular gland fluid. WB, whole blood. n.d., not detectable.
